# Female Genital Mutilation in Sierra Leone: Forms, Reliability of Reported Status, and Accuracy of Related Demographic and Health Survey Questions

**DOI:** 10.1155/2013/680926

**Published:** 2013-09-24

**Authors:** Owolabi Bjälkander, Donald S. Grant, Vanja Berggren, Heli Bathija, Lars Almroth

**Affiliations:** ^1^Division of Global Health, Department of Public Health, Karolinska Institutet, 171 77 Stockholm, Sweden; ^2^Department of Community Health, College of Medicine and Allied Health Sciences, University of Sierra Leone, Sierra Leone; ^3^Department of Health Sciences, Faculty of Medicine, Lund University, P.O. Box 117, 221 00 Lund, Sweden; ^4^Geneva Foundation for Medical Education and Research, 1290 Versoix, Switzerland

## Abstract

*Objective.* To determine forms of female genital mutilation (FGM), assess consistency between self-reported and observed FGM status, and assess the accuracy of Demographic and Health Surveys (DHS) FGM questions in Sierra Leone. *Methods.* This cross-sectional study, conducted between October 2010 and April 2012, enrolled 558 females aged 12–47 from eleven antenatal clinics in northeast Sierra Leone. Data on demography, FGM status, and self-reported anatomical descriptions were collected. Genital inspection confirmed the occurrence and extent of cutting. *Results.* All participants reported FGM status; 4 refused genital inspection. Using the WHO classification of FGM, 31.7% had type Ib; 64.1% type IIb; and 4.2% type IIc. There was a high level of agreement between reported and observed FGM prevalence (81.2% and 81.4%, resp.). There was no correlation between DHS FGM responses and anatomic extent of cutting, as 2.7% reported pricking; 87.1% flesh removal; and 1.1% that genitalia was sewn closed. *Conclusion.* Types I and II are the main forms of FGM, with labia majora alterations in almost 5% of cases. Self-reports on FGM status could serve as a proxy measurement for FGM prevalence but not for FGM type. The DHS FGM questions are inaccurate for determining cutting extent.

## 1. Introduction

Female genital mutilation (FGM), also known as female genital cutting or female circumcision, is the term used to describe the non therapeutic, surgical alteration of female genitalia which is a traditional practice in mainly African countries [1].

The term “*mutilation*” is controversial and rejected by members of practising communities whose intention is not to mutilate [2]. The term “female genital cutting” has been argued to be more value neutral and nonjudgemental than FGM [3]. It does, however, not accurately describe what has actually taken place, the removal of parts of the body. The name “female circumcision” is often the English translation of the practice from indigenous African languages into English and was commonly used in the 1970s [4]. The term “*female circumcision*” is likely to cause the erroneous comparison with male circumcision which would be wrong both from anatomical and religious aspects. 

The WHO and the Inter-African Committee on Traditional Practices Affecting the Health of Women and Children (IAC) have adopted the term “female genital mutilation” because not only is it used as an effective policy and advocacy tool [3] but also is a more apt description of the physical act and extent of injury on the genitalia when the procedure is performed [5]. Thus, whilst recognising that the intention of practising communities is not to mutilate, the term FGM indicates the harm and damage caused by the practice [6].

In this paper, the term FGM is used to emphasise that although in most cases in Sierra Leone the intention is to satisfy traditional and cultural reasons, the effect of the practice remains mutilation. In addition, the nature of the paper is original research on the different forms of FGM that are present in Sierra Leone.

FGM is performed mainly in Africa as well as a number of countries in Asia and the Middle East [7]. Whilst worldwide estimates of women who have undergone FGM vary from 130–140 million [8], recent estimates indicate that FGM occurs in 27 African countries affecting 67.7 million girls and women aged 15–49 [9]. This number rises to 85.9 million women aged 50 and older who have undergone FGM in the 27 African countries [9]. An estimated 3 million girls are at risk of FGM in Africa every year [10]. Sierra Leone is one of the five countries in Africa where the prevalence rate exceeds 90% for the age 15–49 years and is the only country in southern western Africa with a very high prevalence rate [9].

FGM is a risk factor for several negative health effects. The severity of health consequences of FGM vary considerably and depend on the anatomical extent of the cutting [11, 12].

In the short term, these can include excessive bleeding, local infections, shock, and delay in or incomplete healing [13–15].

Late complications can include scarring, keloid formation of the vulva, genital ulcers and dermoid inclusion cysts, lower abdominal pain, and infertility [16–23].

Studies have also shown that FGM can cause gynaecological and obstetric complications, negative psychological outcomes, and can affect the sexual function of women [24–29].

## 2. Classifying FGM Forms

One of the difficulties associated with providing information on the type of FGM taking place is that clinical verification is required to confirm occurrence of FGM and the extent of the cutting. Then, this data has to be classified. For classification to be consistently accurate, rigorous training needs to be given to both data collectors and the research team for accurate type classification [30, 31].

## 3. WHO FGM Classification

The World Health Organisation (WHO) has classified the forms of FGM into four types [7]. Given the variations in types of clitoridectomy (type I), excision of labia minora and/or majora (type II), and narrowing the vaginal orifice by cutting and appositioning the labia minora and/or majora (infibulation, type III); subdivisions have been created to distinguish between these variations (Table 1). All other harmful procedures to the female genitalia for nonmedical purposes are covered by Type IV such as pricking, piercing, incising, scraping, and cauterization [7].

The WHO classification is important for studies where genital inspections are performed to determine the anatomical extent of cutting. Genital inspections which can yield systemically consistent and reliable data on FGM forms can be limited by cost, knowledge of data collectors, and willingness of respondents to the inspection [11].

A study among girls and women in Sudan which compared the extent of the cutting verified by clinical examination with the corresponding WHO FGM classification found that many respondents who reported they had undergone “sunna” were found to have a form of FGM extending beyond the clitoris, and as many as 39% girls and 54% of the women reporting sunna had actually undergone type III FGM. Not only did the girls and women inaccurately describe the extent of anatomical alteration but also these did not fit into WHO classification [32]. The WHO classification has, since then, been updated with subcategories which make it possible to distinguish between different anatomical alterations. 

## 4. Demographic and Health Surveys

The Demographic and Health Survey (DHS) developed by Macro International (now ICF International) collects data from representative samples of households of adult women (15–49 years) and men to give national representative estimates on demographics, fertility and reproductive health, maternal and child health, and nutrition and knowledge of and practice related to HIV/AIDS [32].

Within the DHS questions, should FGM be a concern in a country, a module of questions on FGM is added to the women's questionnaire, and the answers are used to generate information on FGM prevalence and types for women and their daughters [10]. As well as being asked whether they have heard of FGM and have been circumcised, respondents are asked if their genital area was “*nicked with nothing removed;*” “*something removed,*” or “*sewn shut*” [10].

The responses to these questions generate information on national prevalence rates and types of FGM for the women themselves and for their daughters. 

## 5. Agreement between Reported and Observed Forms of FGM

Knowing girls' and women's FGM status is important for research studies that examine prevalence trends, determinants of the practice, and for evaluating the effects of interventions to address the practice [33].

Self-reporting is the basis for determining FGM status in the DHS and most other surveys. The assumption is that women respond truthfully when asked about their FGM status and that they know what action was performed on the genitalia. It is not possible to provide information from DHS on the accuracy of self-reporting of FGM status as genital inspections are not conducted as part of the survey. 

The possibility that the validity of DHS and other survey responses might be biased is great in situations where legislation and information campaigns are used against the practice [6]. It may be more likely, for example, to imagine that in countries where FGM is outlawed, women may deny their positive FGM status [34].

The self-reported circumcision status of women aged 15–49 was obtained by interview in 1995 in a longitudinal study in northern Ghana. The women were interviewed again in 2000 after the enactment and enforcement of a law against FGM and public campaigns against the practice. The study found that 13% of women who reported in 1995 that they had been circumcised denied that they were circumcised in the 2000 interview, with denial rates as high as 50% in the youngest age group [33]. This study also showed that women who denied being circumcised were significantly younger, more likely to be educated and less likely to practice traditional religion than the women who reported that they were circumcised.

There are relatively few studies which provide information about self-reports and clinical information on FGM status thus making it possible to assess the level of agreement between reported and observed forms of FGM [19, 23, 30, 32, 35–37]. In these studies, the accuracy between self-reported and observed FGM status ranged from 94% in Egypt [35] to 57% in Nigeria [36], suggesting that results based only on self-reporting might be unreliable.

In southwest Nigeria, two research studies examined the accuracy of self-reports of FGM status by also conducting a medical examination and found high levels of agreement of 92% [17] and 79% [30]. It is likely that the level of disagreement between self-reports and examination reflects the respondents' inaccurate knowledge about their status or incorrect examination assessments, rather than women wilfully, wrongfully declaring their FGM status [33], especially as in both studies, they knew that their self-reports were to be corroborated by medical examination. 

## 6. Sierra Leone

Sierra Leone is a country on the west coast of Africa with a population of approximately 6 million people [38]. There are about 14 main ethnic groups, each with its own language, the largest being the Mende in the southeast region and the Temne in the northern region [39]. 

About 11 years ago, the country recovered from a civil war, which renowned for its brutality by rebel soldiers who amputated the arms and limbs of civilians [38]. The third democratic presidential elections took place in November 2012.

## 7. FGM in Sierra Leone

FGM in Sierra Leone is one activity of the initiation ceremony of the Bondo Society, a powerful all women led and run group [40]. Initiation into the Society is a rites of passage ceremony which recognises when a girl becomes a woman in her community. Girls are therefore around the age of puberty when they become members [40].

The initiation ceremony takes place in the Bondo Bush, a private enclosure usually erected several kilometres from the village. The Bushes are run by a Sowei, the traditional woman Bondo leader who also performs the cutting and is responsible for the smooth running of the Bondo Bush whilst it is in session. 

Formerly, the time spent for initiation in the Bondo Bush could take up to a month, but latterly, this period has been reduced to a couple of weeks [40].

FGM is the first act performed, and as the girls heal, they are prepared for marriage and keeping a home as well as taught about the rights and responsibilities of a Bondo Society member [40].

There is a strong connection between ethnicity and the Bondo Society as each ethnic group has its own “Bondo Bush.” So, for example, a Mende girl will attend a Mende Bondo Bush and will not attend a Limba Bondo Bush. There is no national law against FGM in Sierra Leone.

The most common forms of FGM believed to be practised in Sierra Leone are clitoridectomy and excision [40]. No study has been conducted in Sierra Leone which compared self-reported FGM status with genital inspection to validate self-reported FGM status.

This study describes anatomical changes to the genitals following FGM, classifies them using the WHO FGM classification, and compares the findings from genital inspection with reported FGM status and responses from the DHS FGM module.

## 8. Materials and Methods

### 8.1. Data for the Study

The population from which the sample for this study was derived was the controls of a larger clinic-based case control study to determine the association between FGM and obstetric fistula. These controls were matched to cases with fistula, based on ethnicity, age range and number of previous pregnancies, and were recruited from among pregnant women visiting antenatal clinics, ten in northeastern Sierra Leone and one in the main maternal hospital in the capital, Freetown. Pregnant women were recruited because this sample was appropriate for representing a normal healthy population of women and from whom a genital inspection was ethically acceptable.

Six of the clinics were located in town hospitals: Kambia Town Hospital located in Kambia District; Makeni Regional Hospital located in Bombali District; Port Loko Town Hospital located in Port Loko District; Magburaka Town Hospital located in Tonkolili District; and Kono Town Hospital located in Kono District, as well as the Princess Christian Margaret Hospital (PCMH) located in the Western Urban Area of the Capital.

The remaining centres were community health centres in rural areas of the five districts: Rokupr in Kambia District; Binkolo in Bombali District; Mange in Port Loko District; Yele in Tonkolili District; and Tombodu in Kono District. 

All centres were chosen because of the high ANC clinic attendance serving a mixture of urban and rural populations. The percentage of women receiving antenatal care from a skilled provider in the eastern region of Sierra Leone is 89.6%, and in the northern region 81.9% [39].

### 8.2. Preliminary Studies and Sample Size Calculations

Power calculations were performed for the case-control study mentioned above, based on preliminary studies conducted between 2006 and 2007, resulting in an estimation of 300 fistula cases and 600 controls. It was the control sample that was used for this study.

### 8.3. Training of Data Collectors and Supervisors (Including Pretesting of the Tool)

Each participating centre provided at least two members for the research team: at least one woman data collector and one supervisor. The data collectors were selected from different health cadres within the Ministry of Health. These were maternal and child health aides, state enrolled community health nurse, midwives, nurses, and sisters. Supervisors were selected from community health officers, matrons, and sisters. Both data collectors and supervisors received initial training of three days which included research methodology, followed by another seven days during pilot testing. Particular focus was given to data collectors and supervisors being able to recognise and accurately describe the anatomy of the female external genitalia. The interviewing technique and the genital inspection procedure were pilot tested from November 2009 to July 2010 during which 122 completed control questionnaires were collected. These are not included in the present sample. 

The woman had to be pregnant and a first time visitor to the ANC clinic, to ensure that the same attendee was not interviewed twice. Participants were informed of the purpose and format of the study and assured that the data was confidential and that refusal to participate would not compromise care or treatment. Thus, oral consent was obtained prior to the start of the interview, which was verified by the signature on the consent form by the data collector. After consent had been obtained, the trained women health professionals interviewed the participants and performed the genital inspections. The same procedure was used for all respondents independent of age.

### 8.4. The Tool

Data for this study was obtained using a questionnaire which had been used in the Sudan [14] and adapted for use in Sierra Leone.

Participants were asked for their social and demographic details, pregnancy, and childbirth history, as well as their age at which they underwent FGM and their experience of FGM.

Age was assessed on current age and in cases where respondents did not know their age, a year of birth was estimated from additional questions on age at FGM (such as “*How long have you been a member of Bondo?*”), year of marriage, year of first child, or age of first child.

Education was measured from questions on school and level at which schooling stopped.

Religion and ethnicity were determined by open-ended questions which were then coded from a comprehensive list on the interview sheet.

### 8.5. Reporting and Observing Anatomical Description of External Genitalia

Only those respondents who said they had undergone FGM were asked to describe the extent of cutting using the same of questions used by the DHS on FGM [41]. The questions, which were asked one after each other were “*was the genital area pricked?*”; “*was flesh removed?*”; “*was the genital area sewn closed?*” For each question, the response was “*Yes,*” “*No*” or “*Do not Know.*”

All respondents (whether they reported that they had undergone FGM or not) were requested to undergo a genital inspection by the same woman health professional who had administered the questionnaire. The inspector was therefore aware of the participant's self-reported FGM status. 

Data collectors were instructed to describe the anatomy in a structured way. The clitoris, labia minora, and labia majora were observed and recorded if they were fully present, partially removed, or totally absent. Totally absent in this instance meant every visible part of the organ had been removed. 

### 8.6. Ensuring Accuracy and Consistency in the Recording of Genital Inspections

Given the different cadres of health professionals that were used as data collectors, not only were their experiences and skills vastly different but also their health education level and understanding of the anatomy.

We were concerned that if the data collectors did not consistently and uniformly perform the genital inspection and interpret what they saw, this might introduce observer bias into the results. To address this, we developed a systematic approach to genital inspections which all data collectors used, and we also conducted genital inspection training for data collectors on site, during which they were examined in order to be certified that they were using the procedures correctly and consistently for the genital inspections.

### 8.7. The Development and Use of a Systematic Approach to Genital Inspections

A standardised procedures checklist was developed by the Fistula Surgeon and the training team during training which was distributed to all data collectors. This document was used at all sites for all genital inspections.

### 8.8. Genital Inspection Examination and Certification on Site

This consistency training and certification was carried out in the following manner: the fistula surgeon, based at Aberdeen Women's Centre, had worked with and trained all the data collectors who performed genital inspections.

For the controls, training on genital inspection was provided for all data collectors, regardless of their education and experience by the Principal Investigator and the Fistula Surgeon (February 2010). During pilot testing, extensive and regular follow-up training and exercises were conducted on a monthly basis during the site visits. In addition, at the end of the period of pilot testing, the Fistula Surgeon visited each control centre and examined each data collector to conduct a number of genital inspections on site.

The research team noted that the data collectors for the controls were not only more knowledgeable about the anatomy of the genital area but were also more confident and had nurtured good approaches for working with the patients. 

Completed questionnaires were collected once monthly for the first four months and then once every two months thereafter. The data collection period was from October 2010 to May 2012.

### 8.9. Translating Anatomical Descriptions from Genital Inspections to FGM Types Using WHO Classification

In a subsequent activity, the anatomical descriptions were classified into WHO types by two researchers (L. Almroth and O. Bjälkander).

### 8.10. Statistical Analysis

Univariate and multivariate logistic regression analyses were used to calculate odds ratios with 95% confidence intervals, for possible associations between FGM status and independent social variables. Age was treated as a continuous variable, while other factors were grouped into categories as tables show. All variables with *P* < 0.1 in the univariate model were included in the multivariable model.

Statistical analyses were performed using SPSS software.

Ethical permission for the study was given by the Ethics Board in Sierra Leone and the WHO Ethics Board (Review Committee).

## 9. Results

All participants agreed to be interviewed, and the four who refused genital inspection were excluded from the analysis.

A total of 554 females completed both interview and genital inspection. They were aged 12–47 with an average age of 22.4 years. The median age was 21 years, with girls in the 15–19 age range accounting for 44.58% (*n* = 247) of the sample.

Respondents were mainly urban dwellers, married, and from the Temne ethic group. FGM prevalence for the three largest ethnic groups—Temne, Mende, and Limba—is around 80%. The prevalence in the other ethnic groups varies, but due to small numbers, it is difficult to draw any conclusions from these (Table 2). 

Although most participants had been to school, the majority had stopped school at the junior secondary school level, and slightly over a third had never been to school. Main occupations were housewives, traders, and students (Table 2). 

On genital inspection, it was determined that 451 respondents had undergone FGM and 103 had not, giving an FGM prevalence rate of 81.4%. The average age at FGM was 12.6 ± 3.2 years (range 3–22 years). A total of 110 did not know their age at FGM (data not shown).

Table 3 provides results of univariate and multivariate logistic regression analysis for the outcome variable FGM. In both models, increasing number of previous pregnancies, rural residency, religion (Islam), being married, and illiterate are factors associated with higher prevalence of FGM. In the univariate model, there was an association between increasing age and FGM, but this was not significant in the multivariate model. 

### 9.1. Forms of FGM

The form of FGM (using observed anatomical descriptions of genital alterations) and how these correspond to WHO modified typology are presented in Table 4. They show that 31.7% (*n* = 143) respondents had type Ib (removal of the clitoris with the prepuce); 64.1% (*n* = 289) had type IIb (partial or total removal of the clitoris and labia minora); and 4.2% (*n* = 19) had type IIc (partial or total removal of the clitoris, the labia minora, and the labia majora).

Anatomical descriptions of alterations in vulva following genital mutilation show three combinations of cutting extent to be the most common (Table 5). These are “*clitoris absent, labia minora and labia majora present*”—referred to as Combination 1 (*n* = 129, 28.6% of all cutting combinations, type Ib)—“*clitoris absent, labia minora partially removed, and labia majora present*”—referred to as Combination 2—(*n* = 124, 27.5% of all cutting combinations, Type IIb); and “*clitoris absent, labia minora absent, labia majora present*”—referred to as Combination 3 (*n* = 149, 33% of all cutting combinations, type IIb).

Combinations 1 and 2 are twice as likely to occur among those girls living in urban than among girls living in rural settings. Combination 3 had nearly equal numbers of girls from both rural and urban settings. Combination 3 seems favoured amongst the Limba ethnic group compared to the other types, whilst all combinations appear to be in similar numbers for the Mende and Temne ethnic groups. 

### 9.2. Consistency between Self-Reported and Observed FGM Status

During interview, 81.0% (*n* = 449) of participants reported some type of FGM, and 105 reported they had not undergone FGM (19.0%, *n* = 105). 

From the genital inspection, there was evidence of FGM among 81.4% (*n* = 451) of the women, and in 103 cases (18.6%), no evidence of FGM was found on inspection. This means that two women reported that they had not undergone FGM but were found to have undergone FGM on inspection.

### 9.3. Accuracy of DHS FGM Questions

The DHS questions on FGM were asked only of those participants who *reported* that they had undergone FGM (*n* = 449). All participants who reported that they had undergone FGM were found to have had some form of FGM on genital inspection, with slightly over 10% of the respondents stating for each question asked that they did not know what operation had been performed on the genitalia (Table 6).

Table 7 shows the results of observed anatomical description versus respondents' answers to DHS FGM questions. Most participants who said genital area had been pricked had had clitoris entirely removed (seven out of 12). Interestingly, four respondents out of the 12 who said that the genital area had been pricked had had operations on the labia majora.

Participants reported overwhelmingly that flesh was removed from the external genitalia (*n* = 391). This answer, however, included a variety of different alterations to the genitalia, corresponding to WHO types Ib, IIb, and type IIc. 

A total of five participants reported that the genital area was sewn closed. Observed genital alterations in these participants were all forms of type II, and none were of type III as the answer indicates.

## 10. Discussion

For the first time in Sierra Lone, and in this study, information on genital inspections is systematically recorded to determine the typology of FGM taking place. Also, by comparing self-reports of FGM status with clinically determined data on anatomical description, it is possible to make an assessment of the accuracy between self-reported and clinically observed FGM. Given that the questions on anatomical description that the participants answer are exactly those used by the DHS FGM module, this study also assesses the suitability of the FGM questions posed by DHS for determining the forms of FGM.

### 10.1. Limitations

One possible weakness of this study is that the respondents were the matched controls of fistula cases which had been collected for another study based on a matched case-control design. Therefore, the study's sampling procedures are not random, and we cannot state that our sample is representative to women in Sierra Leone in the sense of a random sample. We do, however, believe our sample gives a very good picture of the situation among women in reproductive age in the country. 

We have approached healthy women coming for antenatal care in settings with good antenatal care coverage. Our respondents were selected based on matching women coming for fistula repair to the only two existing centres providing this form of care in the country. Women from all over the country and from all ethnic groups come to these centres. This is important, since ethnicity could have been a predictor of FGM practice in Sierra Leone [40]. The population in this study is more likely to represent girls and women in the population who are at risk to suffer from obstetric fistula. The respondents in this study are mainly from the lower wealth quintiles of Sierra Leone society, and the results do not represent girls and women in higher wealth quintiles.

In spite of the limitations, this study provides a sample that is as close to a representative sample that is practically possible to get and gives a very good description of the present situation among normal healthy women in reproductive age in Sierra Leone.

Our sample is not fully comparable with the SL DHS performed in 2008 [41]. More of our respondents come from urban areas than in the DHS (56% compared to 36%). We have more respondents from the Temne ethnic group (53% compared to 35%) and fewer from Mende (13% compared to 32%). These differences are results of the matching procedure to cases with fistula and reflect the sociodemographic characteristics of women with fistula. 

### 10.2. FGM Prevalence and Sociodemographic Factors

The results on prevalence can provide insights of FGM practices that are taking place now. In this study, 81.4% of women had signs of FGM, indicating that FGM is still widely practised. This proportion is lower than previous studies on FGM prevalence from Sierra Leone Demographic and Health Survey 2008 (SL DHS 2008) and Sierra Leone Multiple Indicator Cluster Survey 2010 (SL MICS 2010) of 93.1% and 88.3%, respectively [41, 42]. This may be because of selection bias caused by matching with fistula cases. This matching may have meant that the sample captured younger than older women attending clinics: 90% of the sample were 25 years old or less. Thus, the older women who are likely to have undergone FGM were no longer either attending ANC clinics [42] or not represented in this sample. 

Previous studies in different settings have shown statistical significance between FGM prevalence and some sociodemographic factors such as age, religion, education, ethnicity, and place of residency [43]. In this study, in the univariate analysis, FGM was significantly associated with age, but this difference was not significant in the multivariate analysis. This may be because age was treated as a continuous variable in the regression model, thus causing a loss of power in the analysis.

An examination of subgroups shows a lower FGM prevalence (74.5%) in the 15–19 age range than any other age range, and this figure is close to the 2010 SL MICS prevalence of 70.1% [42] and the 2008 SL DHS prevalence of 75.5% for the same age range [41]. This proportion may be a reflection of the continued and widespread practice of FGM even in the youngest generation.

However, if the FGM prevalence for the 15–19 age range is compared with the 15–19 age range in other high FGM prevalence countries in West Africa (the Gambia [44], Guinea [45], and Mali [46]), the FGM prevalence for this age range is lowest in Sierra Leone [41]. These results may suggest an emerging abandonment of FGM, and more studies will be needed to investigate this phenomenon in Sierra Leone. 

More girls undergo FGM in the 10–15 age range than any other age range, although there were more girls who were aged between 10 and 15 years during this study who had undergone FGM when they were between 5 and 9 years of age. The next largest group for age at FGM were the Do not Knows (*n* = 110). If we assume that the “*Do not Knows*” did not know when they underwent FGM because they were too young to remember, these results might be an indication that the age at which FGM is carried out now is lower than previously recorded [40]. 

Our results show a significantly higher prevalence of FGM among Muslims compared to Christians (OR 2.1). Even though the groups differ, one can question whether religious belief is actually contributing to or protecting against FGM. A vast majority in both groups still practice FGM, 85% among Muslims and 74% among Christians. In fact, a study in Kersa District in Ethiopia where type of genital cutting was type I (79%) or type II (59%) found a statistical significant association with the Christian religion (*P* = 0.003) [47], whilst a study in southwest Nigeria found women with FGM were found to significantly belong to one Christian religion (Pentecostalism) [48]. From other previous studies and this one, we see that both Christians and Muslims in large proportions practice FGM, even though it is known that FGM is not mentioned in any of the Holy Books. Nevertheless, it would appear that religious belief plays a very important role in the continuation of the practice [7, 30, 49], and the role of religious belief and religious leaders should be considered in interventions against the practice. 

In our study, low education is associated with FGM. This association has also been found true in a study in Ibadan Nigeria among 453 women at antenatal clinics which found that illiterate women were significantly more often positive towards FGM [50]. A similar finding was made in Kersa, Ethiopia, between high FGM prevalence and no education among 858 females of reproductive age [47], and in southwest Nigeria, women with FGM were likely to have received only primary education [48]. Most likely this association has to do with general knowledge and exposure to new ideas and being less dependent on traditional social values and norms.

Interestingly, in this study FGM is associated with previous pregnancies and being married. FGM is sometimes thought of as a prerequisite for marriage [26, 27], which may be one explanation, but these factors may also be a result of coming of age [51, 52]. A cross-sectional study of 1,107 women at three hospitals in Edo State Nigeria noted similar significant associations in delivery characteristics between women who had undergone FGM and those who had not. Women with FGM were younger at first delivery (nearly three calendar years younger, with *P* value < 0.0001) than women without FGM [48].

In the Sierra Leonean context, further research might be warranted that examines whether FGM, a social-cultural factor, influences maternal mortality. A study examining the extent of contributions of sociocultural factors to maternal mortality in seven local government areas in Edo South Senatorial District found that whilst the most relevant sociocultural variables were early marriage/early child bearing, FGM was the third contributing factor after women's decision making power and access to traditional obstetric care services which was shown to significantly affect maternal mortality (*P* = 0.001) [53].

### 10.3. Forms of FGM (Extent of Cutting)

One of the main contributions of this study is the measurement of the extent of cutting. Types I and II account for all forms of FGM found in this sample, with type II accounting for 68% of all FGM. We found no evidence of type III from the genital inspections, although 1.1% of the respondents reported that the genital area was sewn closed. On the other hand, this is much less than 2.6% and 14.7% in 2008 SL DHS and 2010 SL MICS, respectively [41, 42]. A possible explanation for the reported forms of type III might be that the question was not understood properly.

It would appear that there has been little or no change in the forms of FGM practised in Sierra Leone; as in our study, we observed clitoridectomy—where the clitoris is partially removed or completely absent and is type Ib according to WHO FGM classification—in almost one-third of all observed FGM. This finding is similar to a survey conducted in 1985 by Koso-Thomas amongst 300 women in the western area of Sierra Leone where 39% (*n* = 105) had this form [40]. The results are also similar to a study in Nigeria where partial or total removal of clitoris was found in 32.6% of all respondents examined [30]. Similarly in a study which observed type of FGM and possible associated gynaecological and delivery complications in Mali, type Ib was identified in 21% of FGM cases [23]. Our results are however different from observations in Burkina Faso by the same study where type Ib was found in 56% of the cases [23].

In our study, we observed type IIb, in 52% of cases, which is close to the 60% observed by Koso-Thomas [40], and similar to the results from a cross-sectional community survey in the Gambia where 56.6% of respondents examined had type IIb cutting [19]. On the other hand, in Nigeria, the prevalence of this form was only 11.5% [30]. 

The proportion of type IIc cutting, where the labia majora are also affected, in addition to the clitoris and labia minora, is relatively low in our sample. This type of cutting appears to be quite unusual, not only in the Sierra Leonean context but also amongst other neighbouring high FGM prevalence countries in West Africa [44–46].

“Sewn closed” according to the DHS describes when the tissue around the vagina is stitched leaving a small opening for the passage of urine and menstrual blood. It is referred to as type III according to WHO FGM classification and is the most extensive form of FGM. In our study, we do not find any evidence of type III cutting, although three cases (1%) of type III were observed from clinical examination by Koso-Thomas [40]. In the 2008 SL DHS, 2.6% of the respondents reported that the genital area was sewn closed [41] and in the 2010 SL MICS [42] 14.2%. It is hard to make sense of the 2010 SL MICS result, particularly as self-reports were not verified by inspection. Generally, however, our findings as well as those of 2008 SL DHS and Koso-Thomas seem to indicate that type III is not a traditional form of FGM practice in Sierra Leone [40, 41].

### 10.4. Consistency between Self-Reported and Clinically Observed FGM

Another interesting contribution of this study is the assessment of consistency between self-reported and clinically observed FGM status. The questions used to ask women about their FGM status are identical to those used in the 2008 SL DHS [41]. The level of agreement between the responses and the results of the genital inspections was high—99%. Although the study shows that the vast majority of women accurately reported their FGM status (FGM or not), the level of accuracy must be qualified by the clinical and national context in which the study took place. It is possible that women attending the clinic were more likely to accurately state their FGM status, given that they knew they were to undergo genital inspection. 

On the other hand, it could be argued that in the national context of no legislation or public condemnation against the practice, women had no reason to falsely report their FGM status. Thus, this high agreement is quite likely in a country like Sierra Leone where there is public support for the practice of FGM. Although this may have resulted in over reporting of FGM status compared to a country where there was legislation (punitive) against the practice, there was no evidence of over reporting in this study.

### 10.5. Using FGM Questions Asked by the Demographic and Health Surveys to Determine FGM Status and FGM Form

The responses received to the question whether “*Genital Area Pricked*” does not appear to correspond to any genital alteration that was observed. The least genital alteration observed in this sample was partial removal of the clitoris with the labia minora and labia majora intact.

Participants seemed to be clear that “*Flesh was removed*” from the external genitalia (*n* = 391) as most answered this question correctly. Flesh removal appears to have been more extensive in this than any other category as more organs (clitoris and labia minora) were *totally absent* than *partially removed*. 

Given the small numbers of respondents who said that the “*Genital area was sewn closed,*” it is difficult to interpret what this result might mean. However, we note that in two of the five cases in this category, the labia majora had been partially removed.

We interpret the results of anatomical descriptions from the DHS questions as insufficiently precise to determine the form or extent of FGM. The DHS questions are not useful because the answers do not provide enough information on the type of operation that has been performed on the external genitalia (e.g., flesh removed). Those DHS questions which ask about the type of operation do not specify which external genital organ is affected by the operation (e.g., genital area pricked, genital area sewn closed). 

Even if participants accurately report that “flesh was removed” from the genital area, it is not possible to distinguish between types I and II using this response. From a positive response to “genital area sewn closed,” it should be possible to determine if type III is present or not. The results of this study, however, indicate that question is not necessarily understood like that. 

The questions appear to be leading, directing the respondents' answer from the question, and giving rise to a *Yes/No/Do not Know* response which lacks the description needed to capture the nuances of cutting that takes place in countries like Sierra Leone where various combinations of types I and II only predominate. As a result, the information obtained from the DHS responses cannot be translated into different forms of FGM. 

It may be more valuable, for example, to use a diagram of the external female genitalia in these settings and ask the respondents to indicate what operation was performed and what happened to the organ instead of asking the DHS questions. 

Most of the respondents said that when they underwent FGM, their genital area was not pricked, that flesh was removed from their genital area, and that their genital area was not sewn closed, which is a correct description, even though it cannot be used to understand the extent of cutting. Anyway, the women appeared to have a general idea about what had happened to their genitals even though they could not name the exact part of the genitalia which had been removed. 

Anecdotal evidence suggests that respondents would assume from watching the operation being conducted on others in the Bondo Bush, that the same operation was performed on them. Traditionally, the girl is taken to the Bondo Bush for the night. Early in the morning, her face is tied with a piece of white cloth, and she is led to where the operation is carried out. Most girls have no idea of what will take place before this time, nor do they actually see what is removed (*Personal communication with Director, Inter Africa Committee, Sierra Leone, Laurel Bangura, June 2012*). It has also been suggested that “the women do not know during the process but can only say after they have had the opportunity to watch the operation being performed on someone else” (*Personal communication with Director, Amazonian Initiatives Movement, Lunsar, Sierra Leone, Rugiatu Turay, June 2012*). 

## 11. Conclusion

Evidence from this study suggests that forms of types I and II are the most prevalent types of FGM occurring in Sierra Leone today.

Results from this study suggested a declining prevalence of FGM in younger age groups. More studies are needed to understand why these changes may be occurring. These studies should also examine the meaning of the practice to communities in order to encourage the abandonment of the cutting aspect of the tradition.

As the responses on self-reported status of FGM using DHS questions provide an accurate indication of FGM status (yes or no), and it may be possible within certain contexts in Sierra Leone to use self-reporting responses as a proxy measurement for FGM status.

From self-reported anatomical descriptions using DHS questions, however, it is not possible to determine the type of FGM in Sierra Leone. To elucidate the different forms and extent of cutting, a different type of tool is required to capture the anatomical extent of FGM, which would relate more closely to the types of morbidity and complications that might present as a consequence of FGM. To verify the anatomical extent of cutting and type of FGM, it is necessary to perform studies including genital inspection.

## Figures and Tables

**Table 1:** WHO typology, 2007.

Type I: partial or total removal of the clitoris and/or the prepuce (clitoridectomy).	
When it is important to distinguish between the major variations of type I mutilation, the following subdivisions are proposed:	
type Ia: removal of the clitoral hood or prepuce only;	
type Ib: removal of the clitoris with the prepuce.	

Type II: partial or total removal of the clitoris and the labia minora, with or without excision of the labia majora (excision).	
When it is important to distinguish between the major variations that have been documented, the following subdivisions are proposed:	
type IIa: removal of the labia minora only;	
type IIb: partial or total removal of the clitoris and the labia minora;	
type IIc: partial or total removal of the clitoris, the labia minora, and the labia majora.	
Note also that, in French, the term “excision" is often used as a general term covering all types of female genital mutilation.	

Type III: narrowing of the vaginal orifice with creation of a covering seal by cutting and appositioning the	
labia minora and/or the labia majora, with or without excision of the clitoris (infibulation).	
When it is important to distinguish between variations in infibulations, the following subdivisions are proposed:	
type IIIa: removal and apposition of the labia minora;	
type IIIb: removal and apposition of the labia majora.	

Type IV: unclassified: all other harmful procedures to the female genitalia for nonmedical purposes, for example, pricking, piercing,	
incising, scraping, and cauterization.	

Reproduced with permission from World Health Organization. Eliminating female genital mutilation: an interagency statement (UNAIDS, UNDP, UNECA, UNESCO, UNFPA, UNHCR, UNHCHR, UNICEF, UNIFEM, and WHO); 2008.

**Table 2:** Sociodemographic characteristics among 554 women 14–47 years, Sierra Leone, 2010–2012.

Characteristic	No FGM *n* (%)	FGM clinically determined *n* (%)	All respondents
Total	**103**	**451**	**554**
Age			
<14	2 (25)	6 (75)	8
15–19	63 (25.5)	184 (74.5)	247
20–24	24 (18.2)	108 (81.8)	132
25–29	8 (10.9)	65 (89.1)	73
30–34	4 (5.9)	64 (94.1)	68
35–39	2 (8.7)	21 (91.3)	23
40–44	0 (0)	2 (100)	2
45–49	0 (0)	1 (100)	1
Residency			
Rural	56 (23)	187 (77)	243
Urban	47 (15.1)	264 (84.9)	311
Civil status			
Divorced			
Married	46 (12.2)	330 (87.8)	376
Never married	54 (32.3)	113 (67.7)	167
Other	1 (20)	4 (80)	5
Separated	1 (25)	3 (75)	4
Widowed	1 (50)	1 (50)	2
Ethnic group			
Fulah	0 (0)	29 (100)	29
Kissi	1 (33.3)	2 (66.7)	3
Kono	7 (28)	18 (72)	25
Koranko	5 (20.8)	19 (79.2)	24
Krio	2 (50)	2 (50)	4
Limba	13 (17.6)	61 (82.4)	74
Loko	2 (33.3)	4 (66.7)	6
Madingo	1 (10)	9 (90)	10
Mende	15 (20.5)	58 (79.5)	73
Susu	1 (12.5)	7 (87.5)	8
Temne	56 (19.1)	237 (80.9)	293
Yalonka	0 (0)	5 (100)	5
Religion			
Christian	51 (26.2)	144 (73.8)	195
Muslim	52 (14.5)	306 (85.5)	358
Other	0	1	1
Education			
Never been	19 (9.0)	193 (91)	212
Up to primary	11 (11.2)	87 (88.8)	98
Up to JSS	45 (29.2)	109 (70.8)	154
Up to SSS	23 (39)	36 (61)	59
Up to tertiary	4 (13.3)	26 (86.7)	30
Other	1 (100)	0 (0)	1
Occupation			
Farmer	0 (0)	67 (100)	67
Housewife	23 (13.5)	148 (86.5)	171
Student	53 (38.1)	86 (61.9)	139
Trader	12 (9.9)	109 (90.1)	121
Unemployed	11 (28.9)	27 (71.1)	38
Other	4 (22.2)	14 (77.8)	18
Parity			
0	59 (24.2)	185 (75.8)	244
1–3	42 (17.7)	195 (82.3)	237
3+	2 (2.7)	71 (97.3)	73

**Table 3:** Results of univariate and multivariate logistic regression analyses for the outcome variable FGM.

	Univariate			Multivariable		
	OR	(95% CI)		OR	(95% CI)	
Age	1.10	1.05	1.14***	0.99	0.92	1.07
Number previous pregnancy	1.59	1.30	1.94***	1.41	1.03	1.93*
Residency	1.61	1.05	2.47*	1.98	1.21	3.22**
Ethnic group^a^	0.97	0.64	1.49			
Civil status married (Ref.)						
Single	0.31	0.20	0.48***	0.55	0.32	0.93*
Others	0.39	0.10	1.53	0.63	0.14	2.91
Religion Muslim (Ref.)	2.09	1.36	3.21**	2.09	1.28	3.39**
Education illiterate (Ref)						
Primary	0.75	0.35	1.60	1.00	0.45	2.20
Junior Secondary School	0.25	0.14	0.45***	0.48	0.25	0.92*
Senior Secondary School	0.16	0.82	0.33***	0.31	0.14	0.66**
Tertiary	0.68	0.22	2.15	0.97	0.30	3.12

*P < 0.05  **P < 0.01  ***P < 0.001.

^a^Ethnicity was tested both by each ethnic group and Temne compared to all others, both tests not significant.

All variables with *P* < 0.1 were included in the multivariable model.

**Table 4:** Forms and type of FGM derived from observed anatomical description of clitoris, labia minora and labia majora.

Combinations	Frequency per combination	Combination percentage (%)**	Equivalent to WHO typology	Type % of clinically determined FGM
Clitoris, labia minora, and majora all present	103		Uncut	

Clitoris partially removed with labia minora and majora present	14	3.1	Ib	31.7
Clitoris totally absent with labia minora and majora present	129	28.6	Ib	

Clitoris partially removed, labia minora partially removed, and majora present	11	2.4	IIb	64.1
Clitoris partially removed, labia minora absent and labia majora present	5	1.1	IIb	
Clitoris absent, labia minora partially removed, and labia majora present	124	27.5	IIb	
Clitoris absent, labia minora absent, and labia majora present	149	33	IIb	

Clitoris absent, labia minora, and majora partially removed	6	1.3	IIc	4.2
Clitoris absent, labia minora absent, and labia majora partially removed	13	2.9	IIc	

	554***	100		

**Taking 451 (the total number of respondents with FGM) as denominator.

***A total of 554 females completed genital inspection.

**Table 5:** Three most frequent combinations of anatomical description alterations versus sociodemographic factors.

	Combination 1 Clitoris absent, labia minora, and labia majora present Type Ib (*n* = 129)	Combination 2 Clitoris absent, labia minora partially removed, and labia majora present Type IIb (*n* = 124)	Combination 3 Clitoris absent, labia minora absent, and labia majora present Type IIb (*n* = 149)
Age at FGM			
2–4	2 (1.6)	1 (0.8)	0
5–9	12 (9.3)	15 (12.1)	10 (6.7)
10–15	74 (57.4)	43 (34.7)	48 (32.2)
15+	20 (15.5)	38 (30.7)	39 (26.2)
Do not know	21 (16.3)	27 (21.8)	52 (34.9)
Residency			
Rural	43 (33.3)	42 (33.9)	76 (51)
Urban	86 (66.7)	82 (66.1)	73 (49)
Ethnic group			
Fulah	10 (7.8)	9 (7.3)	6 (4.3)
Kissi	1 (0.8)	0	1 (0.7)
Kono	3 (2.3)	5 (4)	9 (6)
Koranko	5 (3.9)	7 (5.7)	5 (3.4)
Krio	0	0	2 (1.3)
Limba	6 (4.7)	19 (15.3)	30 (20.1)
Loko	1 (0.8)	2 (1.6)	1 (0.7)
Madingo	7 (5.4)	0	1 (0.7)
Mende	19 (14.7)	19 (15.3)	13 (8.7)
Susu	2 (1.6)	0	5 (3.4)
Temne	72 (55.8)	63 (50.8)	75 (50.3)
Yalonka	3 (2.3)	0	1 (0.7)
Religion			
Christian	37 (28.7)	48 (38.7)	47 (31.5)
Muslim	92 (71.3)	75 (60.6)	102 (68.5)
Neither Christian nor Muslim		1 (0.8)	0
Education			
Never been	62 (48.1)	44 (35.5)	68 (45.6)
Up to primary	23 (17.8)	24 (19.4)	30 (20.1)
Up to JSS	36 (28)	34 (27)	27 (18.1)
Up to SSS	7 (5.4)	11(8.9)	14 (9.4)
Up to tertiary	1 (0.8)	11 (8.9)	10 (6.7)

**Table 6:** Self-reported anatomical description (using DHS questions).

Anatomical description	Distribution—frequency (%)			
	Yes	No	Do not know	Total
Genital area pricked/nicked	12 (2.7)	373 (83.1)	64 (14.3)	449 (100)
Flesh removed	391 (87.1)	9 (2.0)	49 (10.9)	449 (100)
Genital area sewn closed	5 (1.1)	394 (87.8)	50 (11.1)	449 (100)

**Table 7:** Observed anatomical description versus respondents' answers to DHS FGM questions describing operation performed on external genitalia: area pricked, flesh removed, or area sewn closed.

Anatomical description of clitoris, labia minora, and labia majora	Frequency (%)			
	Genital area Pricked	Flesh removed	Genital area Sewn closed	WHO type (frequency)
Clitoris, labia minora, and majora all present	—	—	—	—
Clitoris partially removed with labia minora and majora present	1 (8.3)	12 (3)	0	Ib (14)
Clitoris totally absent with labia minora and majora present	7 (58.3)	107 (27.4)	0	Ib (129)
Clitoris partially removed, labia minora partially removed, and majora present	0	10 (2.6)	2 (40)	IIb (11)
Clitoris partially removed, labia minora absent, and labia majora present	0	4 (1)	0	IIb (5)
Clitoris absent, labia minora partially removed, and labia majora present	0	111 (28.4)	0	IIb (124)
Clitoris absent, labia minora absent, and labia majora present	2 (16.7)	130 (33.3)	1 (20)	IIb (149)
Clitoris absent, labia minora, and majora partially removed	2 (16.7)	4 (1)	2 (40)	IIc (6)
Clitoris absent, labia minora absent, and labia majora partially removed	0	13 (3.3)	0	IIc (13)

Total	12 (100)	391 (100)	5 (100)	All types (554)
